# Everybody hurts sometimes: perceptions of benefits and barriers in telemedical consultations

**DOI:** 10.3389/fpubh.2023.1223661

**Published:** 2023-07-20

**Authors:** Anna Rohowsky, Julia Offermann, Martina Ziefle

**Affiliations:** Communication Science and Human-Computer-Interaction-Center, Institute of Linguistics and Communication Studies, RWTH Aachen University, Aachen, Germany

**Keywords:** telemedicine, acceptance, perception, benefits, barriers, concerns, care

## Abstract

**Introduction:**

Shifts in the age structure, rising needs of care and support, and a lack of (in)formal caregivers require innovative solutions to relieve the whole healthcare system. Applying digital approaches, such as telemedicine, has the potential to support people in need of care, to relieve caregivers in families and professional environments, and to assist medical professionals in their working everyday life: e.g., using telemedicine for acute consultations could contribute to avoid hospitalizations of older people, whereas consultations with the general practitioner could reduce efforts and relive medical personnel. Beyond technical opportunities and potential, the acceptance of future users represents a prerequisite for a sustainable adoption of such innovative approaches, especially in sensitive contexts such as life in older age in nursing homes.

**Methods:**

This study aimed at collecting users' perceptions and evaluations of telemedicine in nursing homes. Two scenarios of telemedical consultations were applied which were either carried out by an emergency physician in acute situations or by the attending general practitioner. In a first approach, advantages and disadvantages of telemedicine were collected with the help of a qualitative interview study (*N* = 12) with laypersons and medical staff. The identified acceptance-relevant factors were then quantified in a second study using an online questionnaire (*N* = 204).

**Results:**

Outcomes revealed that both types of telemedicial consultations would be gladly used. However, for telemedical consultations in acute situations, the perceived disadvantages outweighed the advantages; for telemedical consultations with the general practitioner, the advantages outweighed the disadvantages. A prominent barrier in both scenarios was perceived impersonality, which limited the willingness to use. Nevertheless, participants indicated that telemedical consultations can be a support for nursing staff.

**Discussion:**

Outcomes may help to derive specific implications and recommendations to develop and realize digital technologies tailored to the requirements, needs, and wishes of diverse stakeholders (i.e., patients, medical professionals) as potential future users.

## 1. Introduction

Demographic change represents one of the global challenges our world is faced with today. The increasing life expectancy caused by development and process in medicine is causing an aging society (1), leading to new problems that need to be faced: If people are getting older, there is an increasing risk of getting chronic diseases. This causes a greater amount of people who need to receive medical care (2). Besides chronic and acute diseases older people are struggling with dwindling capabilities, e.g., motor and sensory problems, leading to very diverse needs of care. In addition, the loss of cognitive strength and resilience can lead to different degrees of support and need of care as well (3). Hence, with an increasing proportion of older people and people in need of care the need for (in)formal caregivers is rising, who are able to support older people with chronic diseases or loss of physical and cognitive facilities. Although there will be more and more people suffering from diseases, there are not enough people to take care of them especially in nursing homes. The count of practicing nurses and caregivers does not cover the need (4).

Therefore, interventions are needed to prevent the nursing shortage from worsening and to train more nurses and caregivers to meet the rising numbers of people in need of care. Digitization and using innovative digital technologies represents an opportunity to relieve formal caregivers and nurses in nursing homes in their daily work, but also informal caregivers within their home environments (5–7). Introducing digitization in form of telemedicine into the health system has the potential to fundamentally change the care system (8). Telemedicine could ensure medical help in acute, preventive, and curative situations without the need of the physician and patient being in the same room. The WHO sees chances in using telemedicine, to create medical support and bridge distances to connect users (i.e., patients, their relatives, medical professionals, and care personnel) (9) representing a great progress for supporting caregivers in their daily work. Besides the technical opportunities of telemedical innovations and approaches, the acceptance of the respective users is of utmost importance for a sustainable adoption and usage within the everyday life. So far, the perceptions and acceptance of telemedical consultations have not been researched in detail. In particular, it has not been differentiated between acute telemedical consultations to avoid hospitalizations on the one hand and telemedical consultations with the general practitioner to relieve medical personnel on the other hand.

Therefore, this study aimed at a detailed investigation comparing the two types of telemedical consultations applying a two-step empirical approach combining qualitative and quantitative methods. Thereby, a first qualitative interview study enabled to identify relevant factors influencing the perception and acceptance of both types of telemedical consultations. A second quantitative study applying an online survey realized a quantification of the acceptance-relevant factors and enabled a statistical comparison of the two consultation options. This paper is structured as follows. First, the theoretical background of acceptance research in general as well as of acceptance research related to digital care in specific is introduced. Then, the empirical design of the two-step approach is presented followed by a description of the main findings. The last section of the paper discusses these findings and highlights the key insights, derived implications as well as limitations and ideas for future work.

## 2. Technology acceptance and telemedicine

This section presents the theoretical background of the present study, starting with fundamental definitions and models within technology acceptance research. Afterwards, previous research on the acceptance of digital innovations in care is summarized.

### 2.1. Acceptance: definition and approaches

Information and communication technologies are becoming more and more popular in healthcare services however, a technology innovation is worth nothing without people who are willing to use it (10). Dethloff (11) defines acceptance as “a positive adoption of an idea, a fact or a product, in the sense of active willingness and not only in the meaning of reactive acquiescence” (p. 18). Acceptance is a fragile good, which is not necessarily given in every patient and in every usage context, it describes an adoption attitude and willingness to use a technology and this process is influenced by a lot of factors, on the personal side of the user, but also the usage context (12). There are many factors that can strengthen or weaken acceptance like cultural frames, individual factors of users, or context. Lucke (13) says that acceptance consists of three components, that build an analytical triangle. At first there is the object of acceptance, here this is represented by the digital innovation in care. This object is determined by all properties and implications that are attributed to it. These are causing associations and reactions. The subject of acceptance is represented by the potential user influencing the own acceptance with individual opinions, experiences, and behaviors. Besides the object of acceptance and the subject, there is a social and cultural frame as well. The so-called acceptance context can change by time and is biased by people, institutions, and conditions with which the subject of acceptance has to deal with. This implicates that acceptance can variate by time and situation, for example changing circumstances and terms (12). Acceptance is also caused by tradeoffs between perceived benefits and barriers seen by potential users of a technology. It is not necessary that disadvantages are not ascribed to a technology. Instead, the perceived advantages must simply outweigh these disadvantages to gain acceptance (14).

Technology acceptance has been empirically described for more than 50 years now and resulted in several theoretical models. The most popular model is the “Technology Acceptance Model” (TAM) by Davis et al. (15) representing the origin of modeling technology acceptance research. It takes two factors, *perceived usefulness* and *perceived ease of use*, and puts them together to predict the adoption and actual usage of technological innovations (16). It represented the basis for many other models, e.g., the “Unified Theory of Acceptance and Use of Technology” (UTAUT) (17) which focuses on user factors influencing the *behavioral intention* and the actual *use behavior*. Beyond that, the TAM has been adapted by a huge range of acceptance models being applied for diverse contexts, e.g., health informatics (18), mobility applications (19), or e-learning approaches (20, 21). The mentioned technology acceptance models have in common that they are limited to very generic factors (e.g., ease of use, perceived usefulness) and do not justice to specific technology-related or context-related parameters. Beyond that, these models are not usefully applicable in research fields in which acceptance factors, such as specific perceived advantages, barriers, requirements, concerns, and needs are not yet identified. Therefore, qualitative explorative approaches are needed in a first step, enabling the missing identification of acceptance-relevant parameters. Only this way, subsequent quantifying studies are usefully realizable.

### 2.2. Acceptance of digital care

The integration of opinions and requirements of diverse stakeholders in the development of software and innovations in the healthcare sector is inevitable in order to achieve an outcome that satisfies all stakeholders with their different viewpoints (22). For this agile development, future patients and all medical stakeholders are extremely valuable in the healthcare sector. However, agile development is a challenging task where several problems (e.g., insufficient empowerment of future users) arise, which is why agile software development is rarely used (23, 24). Nevertheless this is the only way to identify potential barriers or problems and eliminate them before the innovation is introduced.

There has been quite a lot research focusing on perceived benefits and barriers of digital healthcare. Like the technology acceptance models mentioned above point out, introducing a new innovation means to deal with different claims, values, experiences, and motivations of potential users that influence their acceptance. The perceived usefulness of the new technology is a key factor. If the users considered the technology to be useful and appropriate, they were more likely to use it (25). According to Himmel and Ziefle (26) digital devices in medical care were generally perceived useful by potential users. Next to the usefulness or the performance of the new tool, the emotional experiences users make while using the innovation were of importance (27). This could be, e.g., fun using the tool, lived experiences, or told experiences another person made with the tool. Thereby, told experiences trusted people made had a greater influence on the potential users own opinion than the experiences of strangers (28). Potential users see the possibility of a better quality of care and medical supply guaranteed with digital health care tools (29, 30). Besides that, the potential users hypothesized that telemedicine can improve the efficiency of their work. Gabrielsson-Järhult et al. (31) and Rogove et al. (32) found out that there was hope that digital medical technology can compensate missing medical staff likes nurses or caregivers and can reduce costs. Zobair et al. (33) support the assumption that motivation is an important factor affecting the acceptance of telemedicine. In their study, they investigated the use of telemedicine on the basis of a concrete tool used in physician's practices. They found out that staff without a motivation to use the tools perceived them more as a burden then an advantage.

In contrast to these positive aspects, research has also identified several barriers which decreases the willingness to use digital healthcare technologies. First of all, data security and privacy are very important for potential users. For instance, these aspects were the reason why they are not likely to use technologies in the field of ambient assistant living when cameras were integrated (26). Nevertheless, there are some medical problems that could be a motivation to use these technologies despite of these concerns (34). Physicians who treat their patients via telemedical consultations also expressed concerns and perceived disadvantages of the innovation. The distance between physician and patient caused concerns about how effective telemedical consultations really are (35) and if it is still possible to indicate a diagnosis if the physician is not in the same room with his patient and is not able to examine himself. These concerns lead into fear about patients security, their trust toward the physician and the feeling of being overlooked (31, 36). The patients interviewed were not sure about how their symptoms could be communicated and understood by the physician adequately via telemedical consultations (37). In relation to this, there was another disadvantage as the physicians were not able to examine the patients' body. Therefore, there was a greater chance for misdiagnosis. In this case, there was also the question how to deal with it legally. There were various studies that stated telemedicine to be impersonal and more difficult than face-to-face contact of patient and practitioner (29, 30, 38). The physical distance between physician and patient leads to an impersonal relationship (39). Besides this, structural factors as intense costs for a purchase of such innovation or technological-specific factors mentioned by Brauner (40) can strengthen or weaken acceptance.

The concrete use of telemedicine in nursing homes has been investigated too. Telemedicine, in particular telemedical consultations, can be a way to provide continuous care to potential patients in nursing homes. At the same time, hospital admissions can be reduced. However, it is important that the staff is trained as well as possible to implement the technical and medical procedures in an adequate manner (5). Likewise, existing research has already investigated the possible fields of applications for telemedical consultations in nursing homes. Gabrielsson-Järhult et al. (31) found that consultations could be used for minor medical problems to save time and other resources. The use of telemedical applications is not only beneficial for the local staff (e.g., higher satisfaction of work, decreasing workload) but also for the treating physicians (e.g., no unnecessary travel, increasing capacity for treatments) and the residents of the nursing homes (e.g., continuous treatment possibilities, better quality of care) (41). Especially in rural areas, telemedical consultations can in principle enable faster treatment, while in urban regions a connection with specialists is expected to be more timely (42). However, potential problems arise from the constant change of nurses, who have to be trained again and again in telemedical consultations or from technically problems during the consultations (43).

With regard to acceptance in the nursing home environment, (44) found that relatives and potential nursing home residents alike consider using telemedical consultations. Physicians and nursing staff who had already been able to carry out telemedical consultations also felt that the consultations were useful and beneficial for the patients being treated (43).

Summarizing the existing research on digital innovations in care, several perceived advantages and barriers were identified that potentially affect the acceptance. However, there is so far hardly any research focusing on how the situation in which a telemedical consultation is going to be used influences specific perceived barriers and benefits of the innovation. Therefore, we decided to investigate two scenarios in the context of nursing homes. For this purpose, we holistically identified specific perceived advantages and barriers focusing on a comparison for the different scenarios. The combination of the two scenarios results in an all-around view of how telemedical consultations are perceived and accepted from potential users. Medical staff and potential patients were focused as users to get a wide range of answers.

## 3. Empirical design

**Figure 1:**
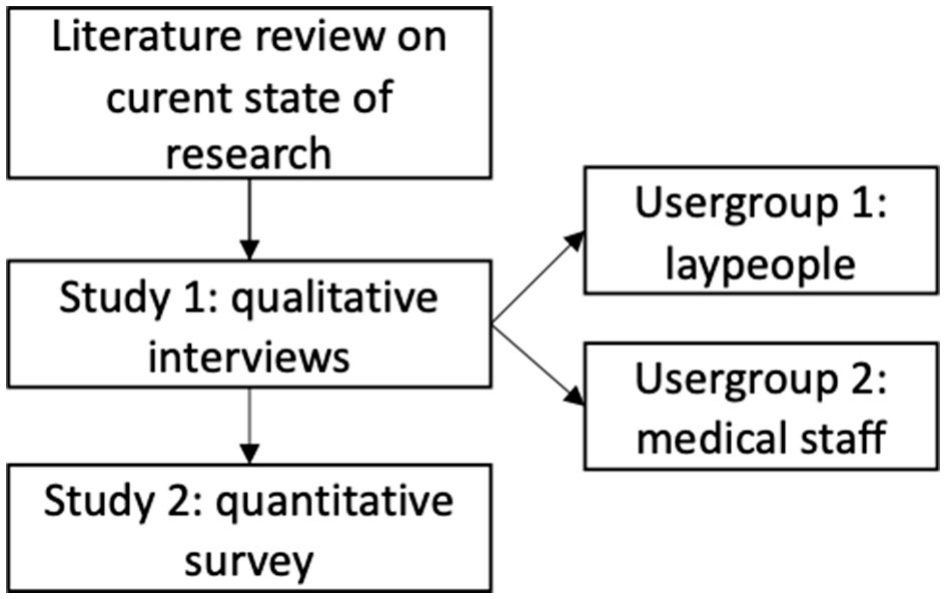
Study design.

As a systematic identification and analysis of acceptance-relevant parameters for telemedical consultations in nursing homes is necessary, we conducted an empirical multi-methodological approach presented in this paper (see Figure 1).

To get a first impression of factors that can influence the perception and evaluation of telemedical consultations or telemedicine in general, we analyzed relevant literature in the field (see Section 2.2). Based on the identified acceptance factors (e.g., perceived benefits and barriers), we conceptualized our qualitative study. We conducted the qualitative interview study and interviewed participants to get a first look into their perceived barriers and benefits of telemedical consultations. The qualitative interview study built the foundation for a subsequent quantitative online survey study where the identified factors were quantified. In combination, these two studies provide a greater understanding of the influencing factors on the perception of telemedical consultations and how important these factors are for potential users.

In both studies, we investigated the perceptions and evaluation of telemedical consultations as one form of telemedicine being applied in nursing homes. Such consultations function as mediators between patients and physicians. Both users can see and hear each other by means of a camera, microphone, and display included in a wheeled stand. With medical devices, that are included as well, the wheeled stand is a possibility to perform check-ups with less personnel effort.

To provide our participants a general understanding of telemedical consultations, we first gave a short introduction to what telemedical consultations look like, how they work and which persons are included in this process. We used the following descriptive scenario, in both the interviews and the questionnaire.

*Please imagine the following: In nursing homes, there is the opportunity to contact a physician with telemedical consultations. For this purpose, there is a wheeled stand in the nursing home. It is equipped with a camera and microphone, so everyone can be seen and heard. There are also many medical devices on this rolling stand, for measuring blood pressure, temperature or pulse. The wheeled stand is pushed in the patient's room. Then a physician is available after a time period of maximum 10 minutes. He will do the check-up with the help of the assistant nurses locally. With a connected program the physician can access the data collected by the wheeled stand and the electronic patient file. The vision is that an online check-up can be sufficient in some cases and avoid a patient being rushed into hospital, instead of staying in their well known environment*.

The telemedical consultation can be imagined in two situations. First in an acute situation that we defined as the following:

*The telemedical consultation can be used in acute situations, where there is an acute medical emergency with a patient or a patient not feeling well at the moment. He can be treated with the telemedical consultation, instead of being taken to a hospital*. For better understanding, we call the telemedical consultations in acute situations *acute consultations*.

The second scenario, the telemedical consultation with a general practitioner is defined as the following:

*Telemedical consultations can be used for general check-ups with a general practitioner, too. This implicates that the patient feels well in the consultation and the check-up is just for routine. With the telemedical consultation the physician does not need to visit the nursing home in person*. In the following, this scenario is called *regular consultations*.

We reminded our participants to empathize with these scenarios when answering the questions of the survey.

## 4. Study 1: qualitative approach

The following sections describe our empirical approach and how we designed the preceding interview study, followed by a description of the interviewed participants.

### 4.1. Methodological concept

We conducted 12 interviews. One of them was done with two participants. All interviews were conducted by the same interviewer. Some took place online and were recorded by the video platform we used for that, but we also did some of the interviews in person and recorded them by phone. The interviews took between 50 and 90 minutes. We transcribed the interviews anonymously and corrected language or grammatical issues if they affected the understanding. We used a mix of the structured and summarizing content analysis (45) and structured the content of the interviews in (sub-)categories. These categories can arise inductive from the interviews' content or deductive on basis of the preceding literature review. We built categories with both methods. First, we figured out which advantages and disadvantages can be found in the existing literature (deductive categories). For this purpose, we were guided by publications that dealt with similar technologies. Then, we analyzed the interviews in relation to these categories to find out to what extent the participants mentioned the same factors. After that, we added the new identified factors and built new (inductive) categories with them or added them to the existing categories. For a better understanding, a definition and an example were specified for every category to differentiate them from another. We did the same process for both scenarios so that two comparable systems of categories evolved. In the subsequent findings Section 4.2, we describe the perceived advantages and disadvantages of telemedical consultations in both scenarios based on the responses within the interviews. In some places, we use direct examples from the interviews for some dis(advantages). These text extracts are meant for illustration and better understanding only. As common for qualitative research, the advantages and disadvantages listed were not only mentioned in these individual examples, but rather reflect the attitudes of the majority of the interview respondents.

#### 4.1.1. Interview design

We designed two interview guidelines that fit to the different user groups (i.e., laypeople, experts: medical personnel and physicians). We adapted the scenarios in wording that they fit to the user groups. First, every participant was informed about data security and privacy. Subsequently, the first part of the interview contained some questions to get to know the demographics (e.g., age, gender, and occupation). Furthermore, we asked the participants about their experience with assistance and care of older people. If they had expertise with it, they were asked to explain their experiences and how they perceived care in general. Additionally, the participants were asked to tell us about their handling with technology and unknown situations to find out more about their technology affinity and how open minded they are. Besides, the participants were asked to assign either the attribute risk friendly or security minded to themselves.

The second part of the interviews focused on providing information about telemedical consultations. The participants were informed about telemedical consultations and their application, potential, and implementation in nursing homes. We used a video to underline the functionality, so the participants were able to imagine how the hardware could look like. The video showed a prototypical idea of a rolling stand and the necessary medical instruments, as well as an acute situation in a nursing home, in which telemedical consultations could be applied. The demographic change was also explained in order to give the participants an understanding of the overall challenges. Based on that, we first introduced telemedical consultations used for acute situations based on the previously described scenario (see Section 3). The participants were asked to tell their first associations. After that, we wanted to know about perceived advantages, disadvantages, and concerns the respondents have concerning the consultation in the acute scenario. We then asked them about the courses of action they would like the attending physician to take. We also wanted to know about no-gos and must-haves. In conclusion, the participants were asked to rate the acute consultation with a school grade from 1 to 6 (1, best rating; 6, worst rating).

The next part of the interview dealt with the regular telemedical consultations with a general practitioner. The questions we used to understand the perception of this scenario were the same as in the second part of the interview, mentioned before. Hence, the participants shared their opinions regarding (dis-)advantages and concerns, courses of action of the physicians, and a final assessment of general, planned telemedical consultations.

At the end the participants were able to provide feedback and comments on the interview and technology. The participants that can be allocated to the user group of physicians had two extra questions in both scenarios. We asked them about their perception of being able to treat the patients adequately with the help of the telemedical consultations and wanted to know if there are any courses of action the supporting medical personnel needs to have. An exemplary interview guideline can be found in Appendix 1.

#### 4.1.2. Sample description

We conducted 12 interviews, with 13 participants in total. The youngest participant was 26 years old, the oldest was 88 (M = 51.2; SD = 18.5). Six participants were male and eight were female. Six of the interviewees can be allocated to the user group of medical staff, including the physicians. The other seven participants were laypeople. The laypeople had at most private experience with assistance and care of older people and no medical education. Most of the interviewees told us to like working with new technologies and integrate them in their daily life. They all said that they feel healthy even if some of the participants had chronic diseases. All the participants that had experience with assistance and care said, that the medical and care personnel seems to have a very hard job, that is physically and psychologically burdening. Everyone saw the need for more effective and efficient ways to support the workers.

### 4.2. Qualitative findings

The participants discussed numerous advantages and disadvantages of telemedical consultations, from which the most relevant aspects are mentioned in the following. As it can be seen in Table 1, the interviewees discussed benefits of telemedical consultations in acute situations that can be sorted in three categories. The first category united benefits *relevant for patients*, that are treated trough the telemedical consultations. Participants mentioned that there is no waiting time before the consultations can be used to talk to a physician, like there would be if the patients is taken to a hospital. Hence, the patient can be treated faster.

**Table 1:** Perceived benefits of telemedical consultations in acute situations.

**Main category**	**Subcategory**
*Relevant for patients*	No waiting times
	Reducing stress
	Centered to the patient
*Relevant for personnel*	Better work efficiency
	Facilitating the work of medical personnel
*General advantages*	Time efficiency
	Avoiding unnecessary hospitalizations
	Saving resources
	Supplying rural areas

“*Also for patients, very often waiting in the hospital. They really experience it that way... they are usually traumatized. You send them to the hospital and they come and say they won't go back because they waited for five hours, no one was interested in them and there are enough cases like that.”* (General practitioner, 47)

Besides, the acute consultation has the potential to reduce stress, because the patients can stay in their known environment and can be treated by known personnel. Participants also think acute consultations are centered to the patient. Wishes and well-being of the patient are perceived as paramount. It is also perceived that the acute situations can be assessed very good, because the consultations can be done with known physicians.

The next category included advantages being especially *relevant for personnel* that uses acute consultations. The participants thought of a better work efficiency as more emergencies can be treated at the same time. Furthermore, the consultations were perceived to facilitate work as hospitalizations imply high workloads for medical personnel, which can be avoided by using telemedical consultations in acute situations. In this regard, most of the participants thought that the relief outweighs the effort.

The interviewed participants also saw *general advantages* of acute consultations. First of all, resources, such as personnel and costs can be saved. Beyond that, medical supply in rural areas is ensured where emergency staff can barely help. Acute consultations are not seen as a replacement of ambulances and emergency physicians but could support them in rural areas. Time efficiency is also an advantage perceived by the participants. It implicates that the patients get faster help and the treatment can be done faster without waiting for an ambulance or physician. More patients can be treated in short time. In addition to that unnecessary hospitalizations can be avoided, which obtains the capacity of hospitals for treatments of real emergencies and reduces patients' stress and staffs workload. The following statement highlights a combination of the mentioned general advantages:

*These unnecessary operations are simply avoided. Often its such small things that everyone panics and then you look at them or say, Okay, its clearly this and this, and these are very often such unnecessary missions, especially in nursing homes*. (Emergency Physician, 56)

Participants also thought of barriers (Table 2), they had by empathizing with using acute consultations. These barriers can be structured in three categories. The first category is about disadvantages being *relevant for patients* treated by acute consultations. Participants pointed out that communication with the physician is perceived as impersonal and lacking empathy. They justified this with the purely digital contact. In this context, an empathetic conversation is not possible to the same level compared to contact with a physician on a face-to-face basis:

**Table 2:** Perceived barriers of telemedical consultations in acute situations.

**Main category**	**Subcategory**
*Relevant for patients*	Impersonality
	Loss of time
*Relevant for personnel*	Complex usage
	No physical examination
	No overall impression of the patient's status
*General Disadvantages*	Missing infrastructure
	Technical failure
	Missing technological understanding of personnel

“*The personal contact, sure, it's something else to contact someone via the screen. Non-verbal aspects get lost and I don't know, when it comes to critical emotional situations, sometimes a hand on the shoulder is also medicine.”* (Physician, 33)

Besides, the acute consultations can also cause a time loss, if an ambulance is needed against expectations. This could have serious consequences for the patients.

The next category of barriers referred to aspects being *relevant for personnel*. Participants mentioned the perceived complex usage of the technology as a problem and a reason why it is probably not used as it should in stressful situations. The second barrier in this section was related to the fact, that no physical examinations can be done by the physician. He has to rely on the measurements done by the wheel stand and the local personnel. Appropriately, the participants expressed that the physician is not able to gain a full impression of the patient, because of the digital contact.

Finally, there were some *general disadvantages* as well. There was skepticism about the missing infrastructure, especially in rural areas, which is needed for using the acute consultations, i.e., if there is no safe infrastructure like a well working internet connection, acute consultations cannot be conducted. While performing the acute consultations there is a chance of technical failure, which can cause serious problems and missing help for the patient:

“*If something breaks down, the physician is not on the way, nor is the ambulance, and I'm lying there and no one can help me. Some things are important in acute cases.” (Office clerk, 58)*

Furthermore the participants were worried about the technological understanding of the personnel, which is perceived as insufficient, to understand how the consultations work and what to do.

Moving to regular consultations, the participants expressed disadvantages and concerns, too. All disadvantages for regular consultations can be seen in Table 3 and do not differ greatly from the barriers in these categories for the acute consultations.

**Table 3:** Perceived barriers of telemedical consultations with a general practitioner.

**Main category**	**Subcategory**
*Relevant for patients*	Impersonality
	Invasion of privacy
	Too fast handling of the examination
*Relevant for personnel*	Complex usage
	No physical examination
	No overall impression of the patient's status
*General Disadvantages*	Missing infrastructure
	Technical failure
	Missing technological understanding of personnel

As one example for mentioned barriers *relevant for patients* and as mentioned before for the acute consultations, the perceived impersonality was also a problem in regular consultations. Besides participants experienced the regular consultations as an invasion of privacy of the patients, that are treated in their own rooms and seen by camera. The monitoring of the patients in their familiar surroundings was seen as an unwanted intrusion. Furthermore, the participants were worried that the examinations are handled too fast and the physician does not take enough time for their patients.

Participants thought of several benefits of regular consultations in *general* as well as related to the *patients* or *personnel* (see Table 4). First and *relevant for the patients* no waiting time for patients was mentioned, because the consultation can be done where the patient is at the moment. Furthermore, more regular check-ups can be implemented, which is currently not necessarily given in nursing homes. This is caused by the gained flexibility of the physician that makes it easier to treat more patients in less time. The missing travel time to the patients fits in there, too:

**Table 4:** Perceived benefits of telemedical consultations with a general practitioner.

**Main category**	**Subcategory**
*Relevant for patients*	No waiting times
	Regular check-ups possible
*Relevant for personnel*	Flexibility of the physician
	No travel time for the physician
*General Advantages*	Low risks of infections
	Supplying rural areas

“*At the very least, he saves on travel time, which is certainly something that has a time component.” (Marketing Manager, 49)*

A perceived *general advantage* of the regular consultations was the low risk of infection with flue or else, because no germs were taken in nursing homes. Besides the participants mentioned the possibility of providing better medical care to rural areas, as seen for acute consultations.

## 5. Study 2: quantitative approach

In the following section, the quantitative study will be presented. First, we will deduce our hypotheses from the qualitative findings. Secondly we describe the applied study design and the characteristics of the sample. Finally, we present our quantitative findings.

### 5.1. Research questions and hypotheses

The findings and the impressions of the qualitative studies enable to derive and define research questions and hypotheses for the subsequent quantitative study.

First of all, the identification of specific perceived benefits and barriers provide the basis for a quantification of the acceptance-relevant factors differentiating between telemedical consultations in acute situations and with a general practitioner. Therefore the first research questions focus on the quantitative evaluation of benefits and barriers.

1. RQ1: What are the most relevant specific benefits and barriers of telemedical consultations for acute situations?
2. RQ2: What are the most relevant specific benefits and barriers of telemedical consultations with a general practitioner?
3. RQ3: Are there differences in the evaluation and perception of the two types of consultations?

Besides the identification of relevant advantages and disadvantages (RQ1, RQ2), the qualitative study enabled indirect comparisons between both types of consultations which are described here in summary. To elaborate these differences and similarities (RQ3) in more detail, hypotheses are derived based on the qualitative study and the expressed perspectives of the participants.

Overall, the participants' opinions varied greatly with regard to both types of telemedical consultations: Some participants could well imagine using telemedical consultations in acute situations, but vehemently rejected telemedical consultations with a general practitioner. Other participants expressed the opposite opinion, indicating an unwillingness to use telemedical consultations in an acute emergency under any circumstances. Based on these diverse evaluation patterns, it can be assumed that significant differences can be identified for the intention to use both types of telemedical consultations (H1). The advantages of telemedical consultations with a general practitioner were in parts more intensively discussed as the advantages of telemedical consultations in acute situations. Hence, it is assumed and has to be verified that the advantages of telemedical consultations with a general practitioner are perceived and evaluated better than consultations in acute situations (H2). In line with this, some participants expressed a more critical perspective on potential disadvantages of consultations in acute situations. Therefore, it is assumed and has to be clarified, if potential disadvantages of telemedical consultations in acute situations are perceived and evaluated worse than disadvantages of consultations with a general practitioner (H3).

In general, the interviews showed a higher relevance of perceived advantages compared to disadvantages related to both types of telemedical consultations. Therefore it is assumed that the perceived advantages prevail the perceived disadvantages for using telemedical consultations in acute situations (H4) as well as for using telemedical consultations with a general practitioner (H5). A final conclusion of the participants showed again diverse evaluation patterns: some participants expressed to perceive telemedical consultations with a general practitioner to be more useful and beneficial, while few preferred telemedical consultations only in acute situations to increase security. Therefore, it is assumed that overall telemedical consultations with a general practitioner are evaluated and perceived better compared to telemedical consultations in acute situations (H6).

These hypotheses are summarized in the following:

1. H1: The intention to use differs in both scenarios.
2. H2: The advantages of the telemedical consultation with a general practitioner are perceived better than the advantages of the telemedical consultation in acute situatons.
3. H3: The disadvantages of the telemedical consultation in acute situations are perceived worse than the disadvantages of the telemedical consultation with a general practitioner.
4. H4: The advantages of the telemedical consultation in acute situations prevail the disadvantages.
5. H5: The advantages of the telemedical consultation with a general practitioner prevail the disadvantages.
6. H6: Telemedical consultations with a general practitioner are perceived better than the telemedical consultation in acute situations.

### 5.2. Methodological approach

In the following, the methodological approach of the quantitative study is presented, describing the design of the online survey, data measurement and analysis as well as the characteristics of the sample.

#### 5.2.1. Questionnaire design

**Figure 2:**
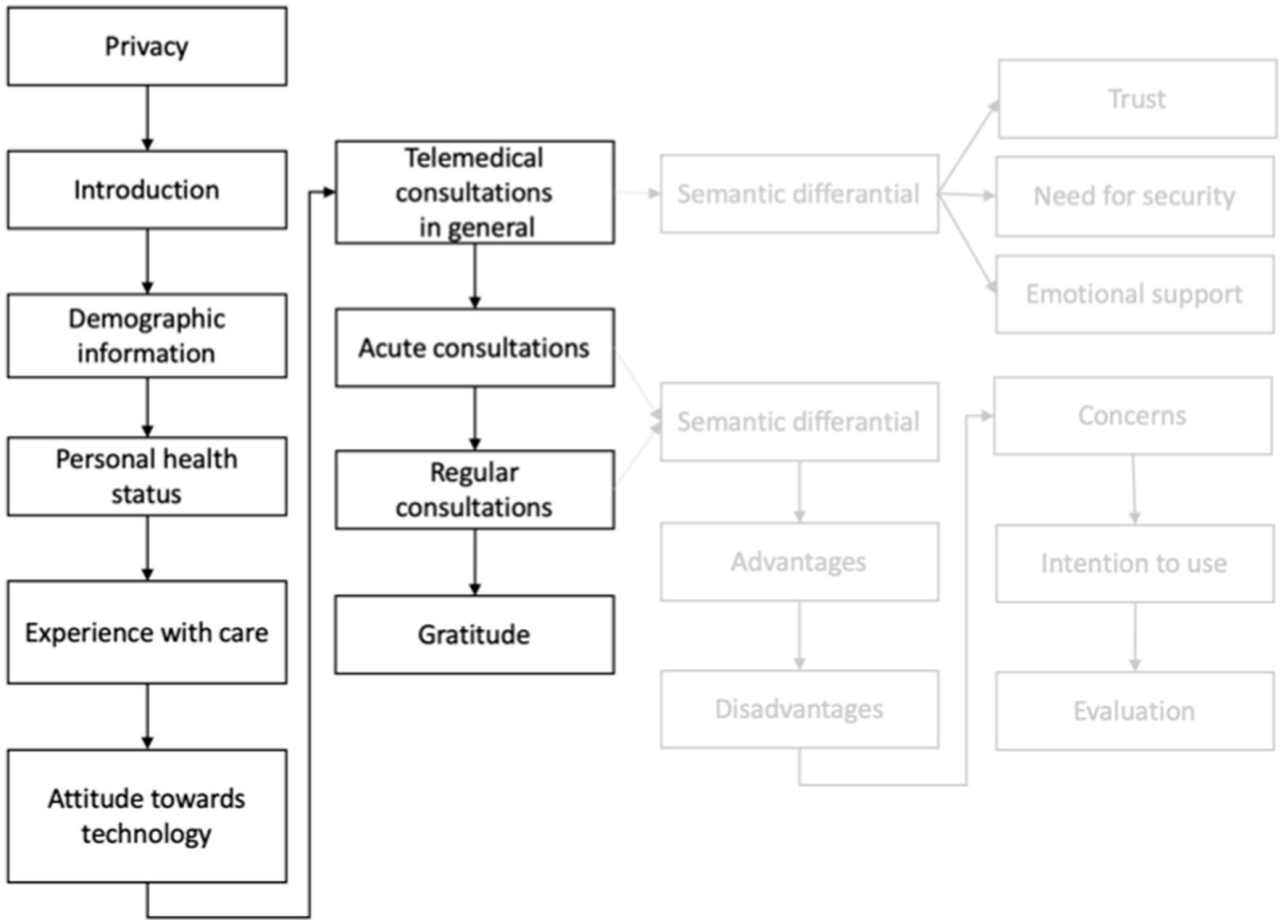
Structure of conducted survey.

The applied questionnaire design is presented in Figure 2. The dark gray part shows the sections of the questionnaire. The lighter gray parts indicate the exact structure of these sections. The two sections, where the scenarios were retrieved, were structured the same way.

After an introduction to the topic and information about data security, the first part of the questionnaire asked for demographic data of the participants (i.e., age, gender, and educational level). Further, the participants evaluated their health status and provided information about their experience with care. Besides, we wanted to know about the participants attitude toward technology measured by four items (see Table 5). After that, we introduced the concept of telemedical consultations and asked about the participants' general opinion and intention to use.

**Table 5:** Results of reliability analyses of the investigated constructs.

**Scale**	**Items**	**Cronbach's alpha**
Affinity for Technology Interaction (ATI)	4	0.816
Benefits of acute consultations	9	0.906
Barriers of acute consultations	8	0.845
Benefits of regular consultations	6	0.839
Barriers of regular consultations	8	0.862
Intention to use for acute consultations	3	0.925
Intention to use for regular consultations	3	0.915

The next two parts of the online questionnaire were the same, focusing on the evaluation of the specific types of consultations, and related either to telemedical consultations in acute situations or to telemedical consultations with a general practitioner in a randomized order. For the descriptions of the consultations, we applied the descriptive scenario texts we also used in our qualitative study.

For each scenario, the participants assessed perceived advantages which were previously identified in the qualitative interview study (measured by nine and six items, see Table 5). In line with this, the participants also evaluated perceived barriers related to the use of both consultation types (for each type based on eight items, see Table 5). Subsequently, the participants also evaluated their intention to use each of the consultation types (based on three items, see Table 5, e.g., “I would like to use telemedical consultations”). At the end of the evaluation of both scenarios, the respondents assessed their final impression of the two types of telemedical consultations from 1 to 5 (min = 1: very worse; max = 5: very good).

#### 5.2.2. Data measurement and analysis

The results of reliability analyses of all evaluated constructs can be found in Table 5. The measured items referring to the evaluation of telemedical consultations were assessed on six-point Likert scales (min = 1; max = 6), whereas the value of 3.5 represented the mid-point of the scale. Hence, values < 3.5 indicated rejection, while values >3.5 indicated acceptance of an item. The summarizing assessment was measured by a 5-Point-Scale which was shown by an emoji which could switch face expressions (min = 1: very worse; max = 5: very good). The level of significance was set at the conventional level of 5%, and values above the significance level (*p* > 0.05) were interpreted as not significant (n.s.).

To describe our sample and results we used descriptive statistics like means (M), standard deviations (SD), and relative frequencies (%) and compared them on a basic level. For testing the hypotheses, we conducted paired t-tests to compare the evaluations of telemedical consultations in acute situations and telemedical consultations with a general practitioner. In more detail, t-tests compare means of two groups, when doing a paired t-test these groups are found in one participant e.g., different times, or like in our study, with regard to different different scenarios. If the difference between groups is not incidental the results turn significant. We used SPSS Statistics (version 28.0.0.0) and JAMOVI (version 2.3.18) were applied as software for the conducted analyses.

#### 5.2.3. Sample description

The questionnaire was completed by *N* = 204 participants. The participants were between 18 and 74 years old (M = 34.7, SD = 15.9). 81.4% (*n* = 166) identified as female, 17.6% (*n* = 36) as male and 1.0% (*n* = 2) as diverse. Most participants (38.7%, *n* = 79) indicated to have a university degree. 36.8% (*n* = 75) reported to have a university entrance qualification. Most of the participants indicated to be employed (40.2%, *n* = 82) or to be a student (40.2%, *n* = 82). Furthermore, almost all participants evaluated their health status positively with perceiving it as rather good or better. The technology affinity was on an average level (M = 3.61; SD = 1.02). Only 18.6% (*n* = 38) of the respondents have experienced care on a professional level, because it's their field of work. 25.5% (*n* = 52) have been actively taken care of someone. 49.5% (*n* = 101) of the participants experienced care indirectly because of a family member or another person, that needed care.

### 5.3. Quantitative findings

In the following, the findings of the quantitative online survey study are presented answering the underlying research questions.

#### 5.3.1. Evaluation of telemedical consultations in acute situations (RQ1)

**Figure 3:**
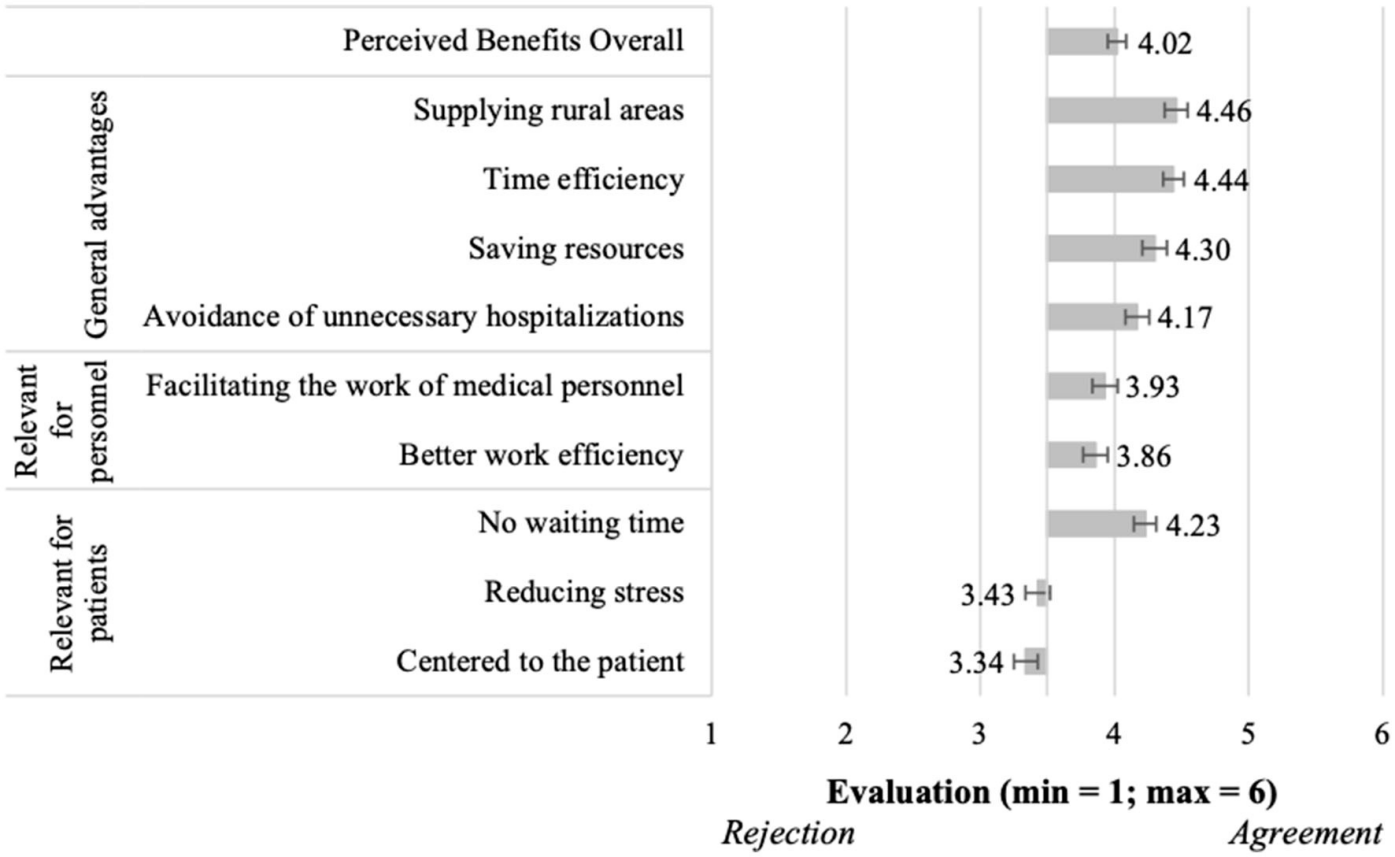
Perceived benefits of using telemedical consultations in acute situations (*N* = 204).

**Figure 4:**
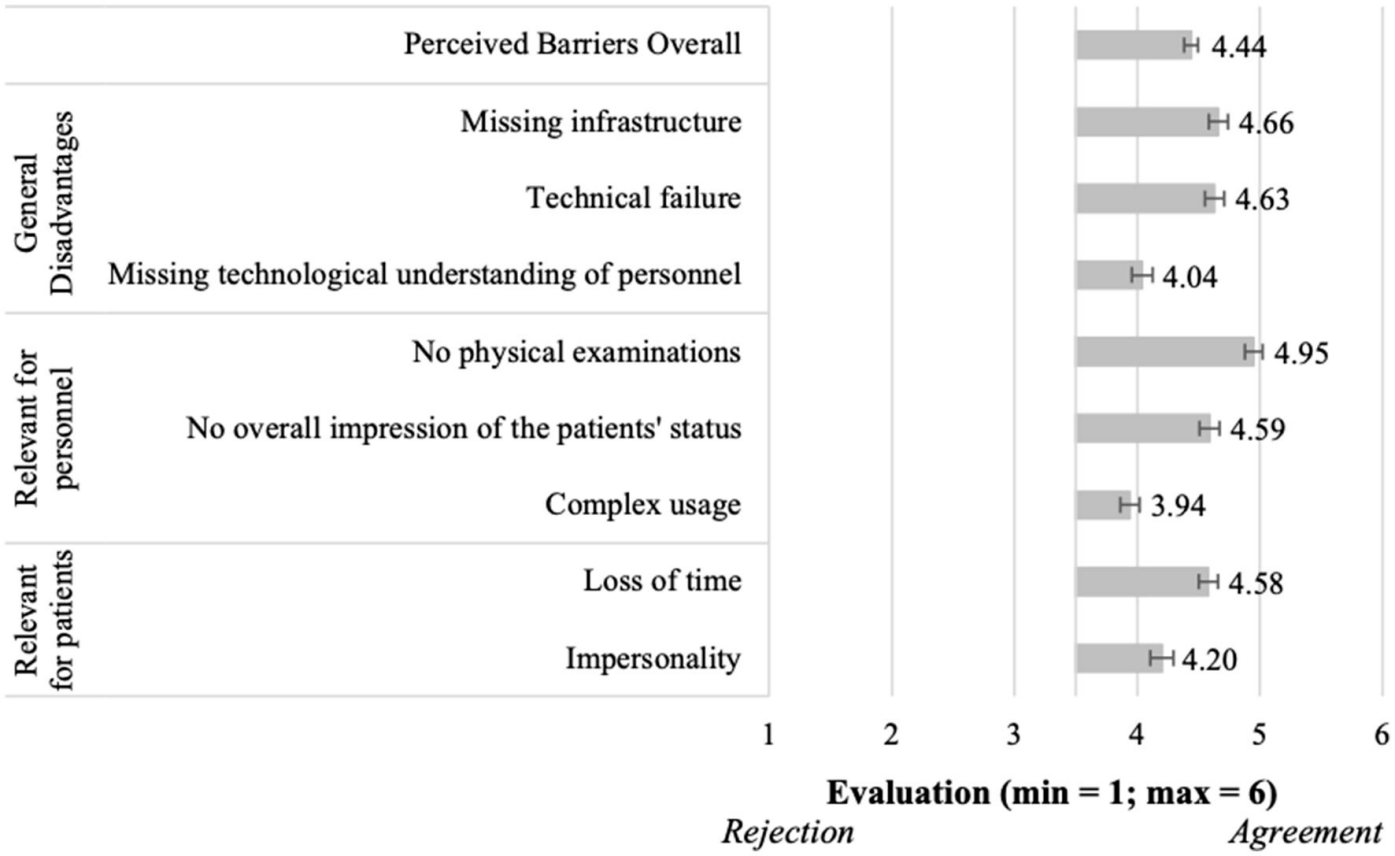
Perceived barriers of using telemedical consultations in acute situations (*N* = 204).

Answering RQ1, the evaluation of perceived benefits and barriers of using telemedical consultations in acute situations is presented in Figures 3, 4. Starting with the overall positively perceived benefits (M = 4.02, SD = 0.95; Figure 3), most single benefits were evaluated with higher means than the middle of the scale, and were thus confirmed to be perceived benefits of using telemedical consultations in acute situations. This was especially true for all benefits referring to *General advantages*, e.g., “supplying rural areas” (M = 4.46, SD = 1.25) or “time efficiency” (M = 4.44, SD = 1.13). Less positively, but still confirmed were the assessments of benefits *relevant for personnel*, e.g., “better work efficiency” (M = 3.86, SD = 1.30). Regarding benefits *relevant for patients*, “no waiting time” (M = 4.23, SD = 1.20) was evaluated most confirmative, while the other benefits were slightly rejected, e.g., “centered to the patient” (M = 3.34, SD = 1.28).

Moving to the barriers of using telemedical consultations in acute situations (Figure 4), the results showed overall also a confirmation of the perceived barriers (M = 4.44, SD = 0.79). Thereby, all single aspects received evaluations higher than the mean of the scale, and thus, all aspects were confirmed to present relevant barriers for the participants. Regarding the *general disadvantages*, “missing infrastructure” (M = 4.66, SD = 1.10) and “technical failure” (M = 4.63, SD = 1.08) represented the most relevant barriers. Among the barriers *relevant for personnel*–and also related to all barriers, “no physical examinations” (M = 4.95, SD = 1.06) represented the most relevant aspect. Further, “loss of time” (M = 4.58, SD = 1.14) represented the most relevant barrier *relevant for patients*.

#### 5.3.2. Evaluation of telemedical consultations with a general practitioner (RQ2)

**Figure 5:**
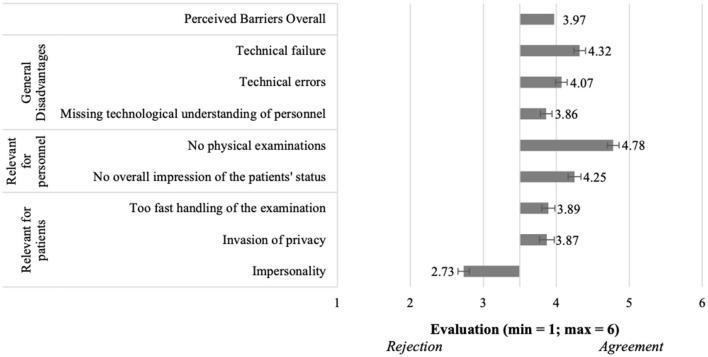
Perceived benefits of using telemedical consultations with a general practitioner (*N* = 204).

**Figure 6:**
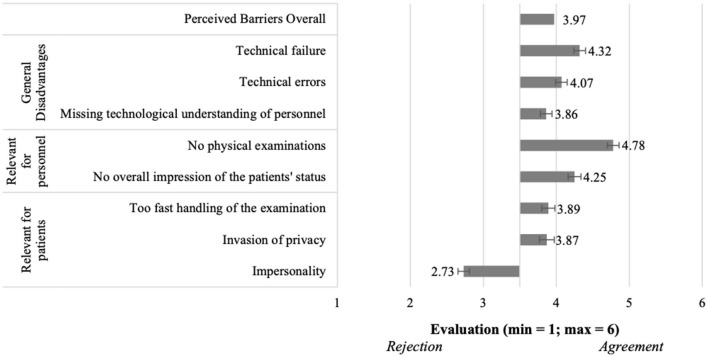
Perceived barriers of using telemedical consultations with a general practitioner (*N* = 204).

Answering RQ2, the evaluation of perceived benefits and barriers of using telemedical consultations with a general practitioner is presented in Figures 5, 6. First, the benefits were perceived clearly positively (M = 4.89, SD = 0.79; Figure 5). Thereby, all single benefits were evaluated with clearly higher means than the middle of the scale, and were thus confirmed to be perceived benefits of using telemedical consultations with a general practitioner. This was true for all benefits referring to *General advantages*, e.g., “low risk of infection” (M = 5.26, SD = 0.91). The assessments of benefits *relevant for personnel* were also positive, e.g., “no travel time for physicians” (M = 5.24, SD = 0.85). Regarding benefits *relevant for patients*, both items were also positively evaluated, e.g., “no waiting times” (M = 4.53, SD = 1.19).

Moving to the barriers of using telemedical consultations with a general practitioner (Figure 6), the results showed overall also a confirmation of the perceived barriers (M = 3.97, SD = 0.86). Thereby, almost all single aspects received evaluations higher than the mean of the scale, and thus, these aspects were confirmed to present relevant barriers for the participants. Regarding the *general disadvantages*, “technical failure” (M = 4.32, SD = 1.15) represented the most relevant barrier. Among the barriers *relevant for personnel* and also related to all barriers, “no physical examinations” (M = 4.78, SD = 1.08) represented the most relevant aspect. Further “impersonality” (M = 2.73, SD = 1.18) received the lowest agreement (< 3.5), representing the least relevant barrier *relevant for patients* and also with regard to all investigated aspects.

#### 5.3.3. Differences in the evaluation of telemedical consultations (RQ3)

To answer RQ3 we investigated all derived hypotheses (H1–H6) in our quantitative analyses. The results are reported in the following.

**Figure 7:**
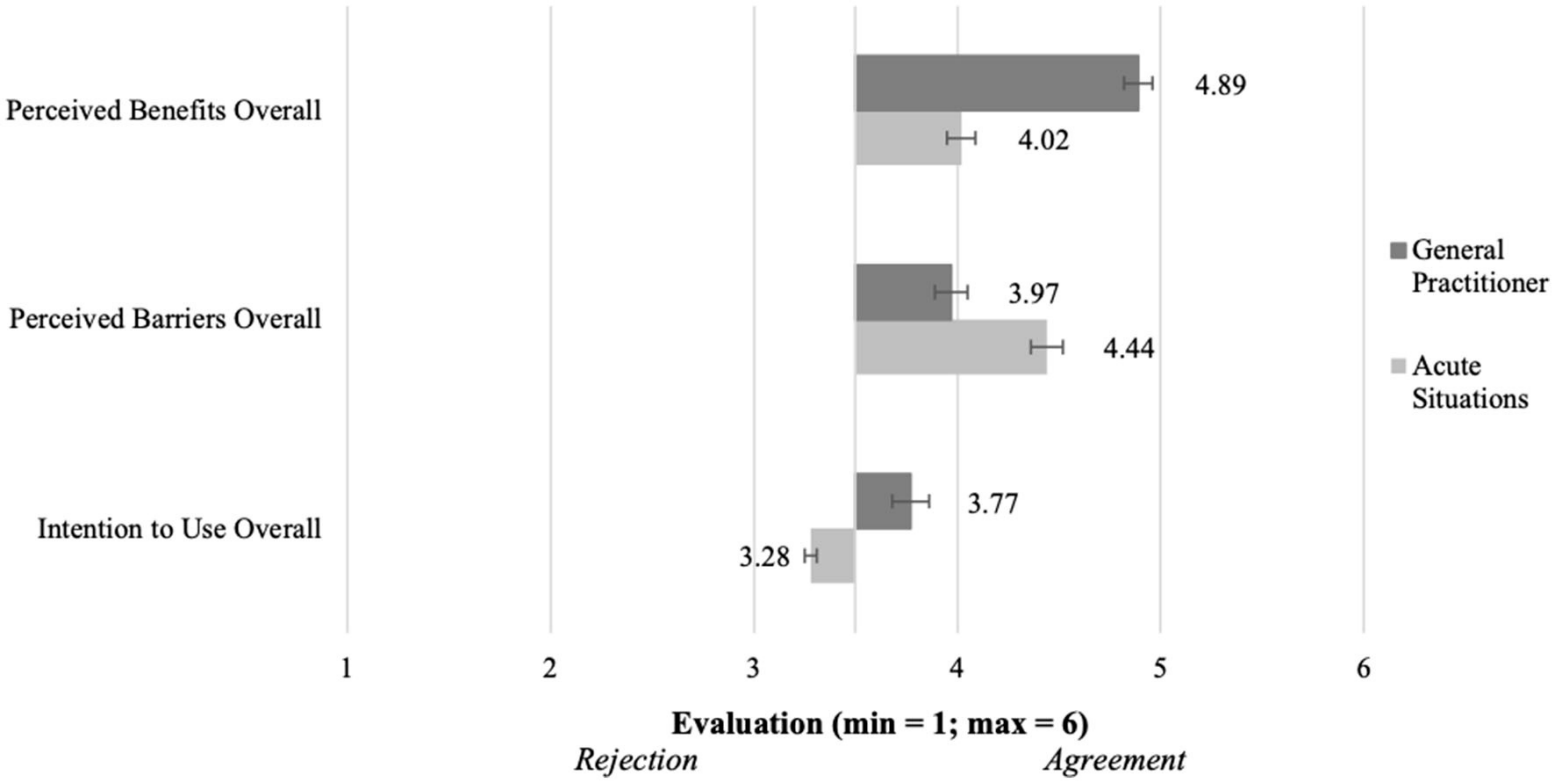
Intention to use, barriers and benefits of the telemedical consultations in both scenarios (*N* = 204).

To test how the intention to use differs in both scenarios (H1), we used a paired t-test. We found out that the intentions to use are significantly different [t(199) = 11.8; *p* < 0.001]. This effect was strong (d = 0.83). As can be seen in Figure 7, the intention to use consultations with a general practitioner (M = 4.34, SD = 1.18) was significantly higher than in acute consultations (M = 3.26, SD = 1.35). Hence, we are able to verify the hypothesis (H1) which postulated a difference in the intention to use.

We combined all perceived advantages for the respective scenarios to get a scale value. To test the difference between both scenarios (H2), we did a paired *t*-test. Verifying H2 (see Figure 7), the results showed that the scenarios differed significantly [t(199) = 14.9, *p* < 0.001]. With a strong effect (d = 1.06), the advantages of the consultations with a general practitioner (M = 4.89, SD = 0.791) were perceived more positively than the advantages of consultations in acute situations (M = 4.03, SD = 0.95). Because of the significant difference in the perception of the advantages between the scenarios, we can set the hypothesis (H2) as verified.

We also created scales out of the perceived disadvantages of consultations in both scenarios and tested their difference (H3). The paired *t-*test (see Figure 7) showed that with a medium effect (d = 0.67) the disadvantages of consultations in acute situations (M = 4.45, SD = 0.80) are confirmed more and thus perceived worse that the ones of consultations with a general practitioner (M = 3.97, SD = 0.86). This difference was significant [t(199) = 9.46, *p* < 0.001] and confirmed the postulated difference of the hypothesis (H3).

In order to see whether the participants assigned more value to the advantages or disadvantages of the respective telemedical consultation, the matching variable was evaluated descriptively (H4). It became clear that the disadvantages of consultations in acute situations weigh somewhat more than its advantages (M = 3.11, SD = 1.48 with min = 1 as disadvantages, max = 6 as advantages). Hence the respective hypothesis (H4) can be defined as falsified, as it postulated that the advantages of the telemedical consultations in acute situations prevail the disadvantages. In the case of the consultations with a general practitioner, the participants assigned a higher importance to the advantages than to its disadvantages (M = 4.24, SD = 1.35 with min = 1 as disadvantages, max = 6 as advantages). Therefore, hypothesis (H5) is verified, as it postulated that the advantages prevail the disadvantages with regard to telemedical consultations with a general practitioner.

**Figure 8:**
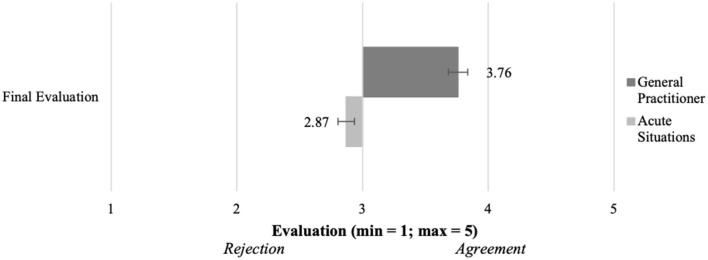
Summarizing evaluation of both acute and regular consultations (*N* = 204).

As shown in Figure 8, consultations with a general practitioner (M = 3.76, SD = 0.96) were seen more positive in the concluding evaluation than consultations in acute situations (M = 2.89, SD = 1.11). This effect was significant and strong [t(199) = 11.0,*p* < 0.001, d = 0.85]. As the respective hypothesis (H6) postulated a more positive perception of telemedical consultations with a general practitioner compared to acute telemedical consultations, the results verify H6.

## 6. Discussion

In our study, we followed a two-step, multi-method approach to find out how telemedical consultations in acute situations and in design as consultations with a general practitioner are perceived and assessed. The interviews showed us, that there are perceived general advantages and advantages for both, patients and medical personnel. The same applies to the perceived disadvantages. The greatest advantage is the reduction of time needed before the patient can be examined by a physician in acute situations. For the regular consultations, the participants mentioned the opportunity for more flexibility of the work of the personnel and physicians and the resulting time savings as best advantages. The most important disadvantage of both scenarios is the perceived impersonality of the contact between physicians and patients. The following quantitative study aimed at an evaluation of the identified aspects. The regular consultation was perceived better than the acute consultation with focus on the perceived advantages and disadvantages. The intention to use was also higher for regular consultations than for acute consultations. We will now discuss our findings and set them into the context of current research. Studies on telemedical consultations in exactly these two scenarios are rare, so we fall back to research about general digital medical technology to class our findings.

### 6.1. Key insights

Our study identified and assessed several benefits and barriers for acute consultations (RQ1). The most relevant ones were related to three categories “relevant for patients”, “relevant for personnel,” and “general (dis)advantages”. Some identified benefits referred to reducing patients' stress and saving time for both, the personnel and patients. However, it was the opportunity to ensure a better supply of rural areas that was most important for the participants, directly followed by perceived time efficiency. The respondents attributed the greatest importance to the disadvantage of no physical examination by the physician. Answering RQ2 the study also identified and evaluated benefits and barriers of acute consultations. The identified benefits were also put in the mentioned categories and reach from flexibility and time-related benefits to a better supply of rural areas. The most important benefit of regular consultations was the low risk of infection, right before the eliminated travel time of the physician. The barriers were the same as for regular consultations, revealing the missing opportunity to examine the patient physically as most relevant aspect. RQ3 consisted of a conglomerate of six hypotheses. In this regard, all hypotheses were verified except of H4. The results revealed that the intention to use differed for both types of telemedical consultations, while the willingness to use regular consultations was higher (H1). In line with this, their advantages were perceived better as the advantages of acute consultations (H2). In accordance with that, the disadvantages of acute consultations were perceived worse than the ones of regular consultations (H3). Our study confirmed that the advantages of regular consultations prevailed their disadvantages (H5), but the disadvantages of acute consultations prevailed their advantages (H4). Altogether the regular consultations were perceived better than the acute consultations (H6).

Overall, some of our results are in line with the existing literature. In this regard, our results confirm that potential patients would give up their privacy in emergency situations (34, 46), but want to protect their privacy during regular examinations, and perceive being filmed as an invasion of privacy (26). In both, the qualitative and quantitative results, special attention is paid to the fact that physicians are not able to perform an examination directly on the patient's body, confirming previous research (35, 37). The basic impression of the patient, which according to the subjects cannot be adequately conveyed to the attending physician, represents a new finding, but fits in content with the lack of physical examinations. In the interviews, the perceived impersonality in both types of telemedical consultations was a major negative factor. However, the feeling of impersonality was perceived as worse in the acute consultations by the respondents of the quantitative study. This raises the question of how the strong difference in the perceived impersonality comes about, since the basic nature of the digital visit remains the same in both scenarios. Here, another factor seems to be crucial for this perception. It could be attributed to helplessness and the magnitude of possible errors in this acute situation, in which all disadvantages are perceived as more influential overall. However, a more detailed rationale cannot be found in this case. (27) already indicates that emotional factors play a role in the evaluation of digital medical technology. Of the investigated factors, impersonality comes closest to such emotionality, yet a further analysis would be useful in order to gain a better insight into influencing factors. While the disadvantages of not being able to be treated directly by the physician and them not being able to gain a holistic impression of the patient are primarily disadvantages for the staff, but this can also have an effect on the patient's sense of security (31, 36, 37). This could explain why these disadvantages are perceived as more serious during acute consultations than during the regular consultations. In an acute emergency situation, patients must be able to rely much more on their attending physicians.

The disadvantages, which were described as general disadvantages, relate, to aspects of the infrastructure that must be created so that the telemedical consultations can be carried out at all. The continuous provision of internet and electricity must be ensured in order to guarantee flawless use. The fear of technical failures or errors is greater in acute consultations than in regular consultations. Here, conclusions can be drawn about the urgency of the situation. Failures during consultations are rated worse in acute situations than in regular consultations. Here it can be said, in line with the explanations already given, that in an acute emergency situation as little as possible should go wrong so that the patient can be medically attended rapidly. This implies that a comprehensive training and examination of the handling of the device and the situation are necessary.

The impression in the interviews that acute consultations are perceived rather less positively by the respondents was supported by the quantitative study. This is shown clearly by both the disadvantages perceived as more negative overall and the advantages of this type of consultation perceived as less positive. This circumstance also seems to have an effect on the intention to use, which is significantly lower for acute than for regular consultations. It is interesting to note, however, that the intention to use regular consultations is still above the neutral value of 3.5 and thus tends to be positive. In contrast, the intention to use acute consultations is below the mean value, i.e., in the rather negative range, which means that this type of consultation tends not to be used. The final evaluation underlines this difference even more strongly and it is not surprising that the final evaluation of acute consultations is significantly more negative than that of regular consultations.

### 6.2. Implications

Some implications regarding the implementation of telemedical consultations can be drawn from the conducted studies. Before the launch of telemedical consultations, it is necessary to clearly triage which cases can be treated in a consultation and which cannot. Especially in acute situations, the fear of losing time if an emergency physician is needed on site is justified. Thus, parameters must be developed according to which the nursing home staff can decide when a consultation is appropriate and when it is not.

As mentioned above, a continuous internet connection and error-free technical functionality must be ensured in order to avoid the occurrence of the aforementioned barriers. In order to establish a basis of trust between doctor and patient, all professionals involved must be adequately trained, especially in the operation of the technology and the conduction of conversations. In order to avoid errors in diagnosis, discussions between nurses–who can perform physical examinations on site—and physicians—who use this information and the available data to make diagnoses—must be practiced and formalized to ensure a smooth process. Special attention should also be paid to the way patients are treated in order to avoid the fear of lack of empathy or impersonality. Doctors and nurses must be actively trained to establish intimacy with the patient, also via digital communication, and to take away the patient's fear of the current situation.

In particular, guidelines need to be established on the criteria that can be used to make diagnoses without the physician being able to perform physical examinations. Legal issues must also be clarified (e.g., who is liable in the event of errors).

In order to implement the perceived advantages of telemedical consultations in the best possible way, a sufficient network of telemedical doctors must be developed to cover both acute situations and regular consultations.

### 6.3. Limitations

The present study applying a two-step empirical approach provided novel insights and enabled an identification and quantification of relevant benefits and barriers for using two different applications of telemedical consultations in nursing homes. Beyond that, our investigation provided first insights into differences of using telemedical consultations in acute situations and for regular consultations with a general practitioner. Nevertheless, the methodological approach and samples of our studies have limitations which should be considered for future research in this field.

Starting with the qualitative sample, the interviewed participants were balanced with regard to age, gender, and professional background. Considering the quantitative study's sample, rather young, predominantly female, and comparably highly educated participants were reached. Future studies should try to reach more balanced samples regarding gender and educational level. As telemedical applications and technologies have the potential to support even older and frail people, future studies should try to focus more on this specific group of future users integrating them into iterative user-centered studies in order to address their requirements, needs, and wishes adequately. Further, the participants originated from one single country–Germany. As health care regulations and supply, but also privacy regulations and perceptions differ strongly across different countries and cultures, future research should try to realize broader samples comparing participants from different countries and origins.

Considering the methodological approach of our studies, the first limitation refers to its scenario-based nature. The applied scenarios representing the different types of telemedical consultations provided the basis for experimentally varying the qualitative and quantitative evaluations of acceptance as well as the perception of relevant benefits and barriers. However, we cannot exclude that evaluations in terms of acceptance or rejection might differ in real-life contexts according to the well-known gap between attitudes and behavior (47, 48). Therefore, future research should aim at a realization of experiments and user studies in everyday life environments (e.g., nursing homes) focusing on hands-on experience with specific telemedical applications. The combination of a qualitative and a quantitative approach was proven to be useful in this field where specific acceptance-related parameters were not distinctly identified before. This way, the qualitatively identified factors were quantified in the subsequent study focusing on two specific contexts of telemedical supply. A further aspect refers to the length of the interviews and the online survey. Due to the two scenarios, the design of the studies may have been repetitive. Hence, a randomized design was applied, in order to minimize the probability of recurrence effects. In this regard, feedback from the participants showed that they understood the reasons for comparing two fundamentally different occasions for telemedical supply. Nevertheless, it is not recommended to compare more than 2 different scenarios comparing the evaluation of different constructs and dimensions. A last methodological aspects refers to the separate analysis of perceived benefits and perceived barriers. The present studies enabled an identification and independent quantification of specific benefits and barriers of using telemedical consultations based on two different occasions. Beyond that, it would be a useful focus for future studies to realize direct trade-offs between potential benefits and barriers of using telemedicine in nursing homes, e.g., by applying conjoint analysis approaches (49, 50). This way, a direct weighting would provide information on when and under which conditions the advantages or disadvantages dominate and are relevant for decisions to (not) use telemedical applications in older age.

### 6.4. Outlook

Research in the field of digital medical technology, especially telemedical consultations, is still ongoing. Further studies are needed to gain a comprehensive impression of the perception and evaluation of these innovations. On the one hand, it is interesting to investigate which other factors manifest trust in telemedical consultations and how they affect the intention to use them. The results of our study show that acute consultations are less popular than regular consultations. For this purpose, it should be investigated which factors also support this perception, apart from the advantages and disadvantages investigated here. If negative factors can be identified, we should then address how to deal with these factors in order to increase trust and willingness to use the services. This could also be done by collecting the terms of use that potential users have of the system and its use. The inclusion of larger samples, both of medical staff and potential patients, is useful here, since both sides should receive an improvement in the acute condition. In order to design the introduction of such technologies, concepts, and guidelines for their use must be drafted. In this context, particular attention should be paid to the issues of data storage, use, and security. Here, qualitative and quantitative studies can generate insights into the fundamental attitudes of potential users toward these issues in the context of telemedical consultations. The interviews gave the impression that the dislike of telemedical consultations is not necessarily caused by tangible factors, but is rather an emotional reaction to the given mixture of circumstances. Perceived impersonality as a specific factor was at the center of the interviews. The existence of two extremes in terms of just that dislike and affection for telemedical consultations should also be further explored. Therefore, it would be interesting to understand what personality factors influence such a perception and what aspects of the telemedical consultations cause it.

## 7. Conclusion

As one opportunity to address challenges caused by demographic change this study aimed at an investigation of the perception and evaluation of telemedicine being applied for support and assistance of care personnel and geriatric patients in nursing homes. The two-step empirical approach of our study enabled an identification of acceptance-relevant parameters and realized a quantitative assessment of these parameters. The special focus in the assessed scenarios on telemedical consultations in acute and regular situations offers insights into the perception of concrete implementation possibilities of telemedical consultations. Here, the results show that acute consultations are perceived more negatively (indicated by lower assessments of advantages and higher assessments of disadvantages) and are less preferred in terms of a lower intention to use than regular consultations. Nevertheless, further studies are needed to gain a broader understanding of factors influencing trust and perception of telemedical consultations, e.g., terms of use, requirements. Especially the field of interpersonal relations shows to be of particular importance in the disadvantages of both scenarios and should be further investigated in addition to possible influences of individual factors (e.g., demographics, expertise) and emotional attitudes.

## Data availability statement

The raw data supporting the conclusions of this article will be made available by the authors, without undue reservation.

## Ethics statement

Ethical review and approval was not required for the study on human participants in accordance with the local legislation and institutional requirements. The participants provided their written informed consent to participate in this study.

## Author contributions

AR: investigation, study design and conception, study conduction, data preparation, data analysis, writing manuscript, and critical review of the manuscript. JO: investigation, study design and conception, study conduction, data preparation, and critical review of the manuscript. MZ: project administration and project funding. All authors have read and approved the final manuscript.
