# Supplementary material for: Everybody hurts sometimes: perceptions of benefits and barriers in telemedical consultations
Source: Front Public Health. 2023 Jul 20;11:1223661. doi: 10.3389/fpubh.2023.1223661 (PMC10399594; doi:10.3389/fpubh.2023.1223661)
Supplement: Supplementary file 1 [file Data_Sheet_1.pdf]

Appendix 1: Exemplary Interview Guideline

These key questions were asked for both scenarios separately: telemedical consultations in acute situations and as regular consultations.

1. What is the first thing you think of when you hear this information about telemedical consultations? Please think of 3 words that come to your mind spontaneously.
2. Off the top of your head, could you imagine receiving care via the telemedical consultations?
3. If you try to put yourself in the situation of a telemedical consultation as a patient, how does it feel?
4. What are the advantages of telemedical consultations in your eyes?
5. Please sort the advantages mentioned according to your perceived importance.
6. What disadvantages do you see with regard to telemedical consultations?
7. Please sort the disadvantages mentioned according to your perceived importance.
8. What concerns do you have when you think about receiving care through telemedical consultations?
9. What conditions would have to be met for you to use telemedical consultations?
10. What information would you need to use telemedical consultations?
11. What courses of action would you like the physician to take?
12. Do you have a must-have and a no-go in mind regarding telemedical consultations?
13. What school grade would you give telemedical consultations?
